# Integrating Blockchain Traceability and Deep Learning for Risk Prediction in Grain and Oil Food Safety

**DOI:** 10.3390/foods15020407

**Published:** 2026-01-22

**Authors:** Hongyi Ge, Kairui Fan, Yuan Zhang, Yuying Jiang, Shun Wang, Zhikun Chen

**Affiliations:** 1 Key Laboratory of Grain Information Processing & Control, Ministry of Education, Henan University of Technology, Zhengzhou 450001, China; gehongyi@haut.edu.cn (H.G.); fankairui@stu.haut.edu.cn (K.F.); zhangyuan@haut.edu.cn (Y.Z.); ws_0702@stu.haut.edu.cn (S.W.); 15237093431@stu.haut.edu.cn (Z.C.); 2 Henan Provincial Key Laboratory of Grain Photoelectric Detection and Control, Zhengzhou 450001, China; 3 College of Information Science and Engineering, Henan University of Technology, Zhengzhou 450001, China; 4 School of Artificial Intelligence and Big Data, Henan University of Technology, Zhengzhou 450001, China

**Keywords:** blockchain, grain and oil food, risk prediction, grey relational analysis

## Abstract

The quality and safety of grain and oil food are paramount to sustainable societal development and public health. Implementing early warning analysis and risk control is critical for the comprehensive identification and management of grain and oil food safety risks. However, traditional risk prediction models are limited by their inability to accurately analyze complex nonlinear data, while their reliance on centralized storage further undermines prediction credibility and traceability. This study proposes a deep learning risk prediction model integrated with a blockchain-based traceability mechanism. Firstly, a risk prediction model combining Grey Relational Analysis (GRA) and Bayesian-optimized Tabular Neural Network (TabNet-BO) is proposed, enabling precise and rapid fine-grained risk prediction of the data; Secondly, a risk prediction method combining blockchain and deep learning is proposed. This method first completes the prediction interaction with the deep learning model through a smart contract and then records the exceeding data and prediction results on the blockchain to ensure the authenticity and traceability of the data. At the same time, a storage optimization method is employed, where only the exceeding data is uploaded to the blockchain, while the non-exceeding data is encrypted and stored in the local database. Compared with existing models, the proposed model not only effectively enhances the prediction capability for grain and oil food quality and safety but also improves the transparency and credibility of data management.

## 1. Introduction

Grain and oil food are essential sources of nutrition in human daily life, with cereals supplying rich carbohydrates and proteins, while oils provide essential fats and energy [1,2]. Ensuring the safety of grain and oil food has a significant impact on national security and the harmonious and stable development of society [3,4]. In recent years, grains have faced multiple risks, including pesticide residues, heavy metal contamination, microbial contamination, and mold during production, processing, transportation, and storage [5,6]. These issues not only jeopardize consumer health but also have the potential to cause social problems and economic losses. Traditional management methods often rely on post-event analysis, making it difficult to achieve early warning and effective response to potential risks. Therefore, it is particularly important to conduct risk prediction for the quality and safety of grain and oil food. It not only helps achieve proactive management and optimize resource allocation but also enhances the scientific basis of decision-making, thereby ensuring the overall safety of grain and oil food [7,8].

Machine learning is an artificial intelligence technology that analyzes large amounts of data to identify patterns and trends for prediction, classification, or decision-making [9,10,11]. In recent years, it has been widely applied in the field of grain and oil food safety risk prediction. Geng et al. [12] developed a food safety risk prediction model using an improved Random Forest (RF) algorithm, which incorporated the Monte Carlo (MC) method to expand the sample data. By generating synthetic samples, the model effectively addressed the limitations of small sample sizes, significantly enhancing prediction accuracy. Experimental results showed that the model outperformed traditional methods, such as Support Vector Machines (SVM), in terms of accuracy, generalization ability, and computational efficiency. Lü et al. [13] proposed a wheat quality safety prediction model based on Extreme Gradient Boosting (XGBoost). The model utilized soil and wheat sample data, applying multiple machine learning algorithms and incorporating SHAP analysis to identify soil Cd content and pH as the key factors affecting wheat Cd concentration. The results indicated that the proposed XGBoost model achieved outstanding predictive accuracy in wheat quality safety prediction. Zhang et al. [14] proposed an intelligent food safety risk early warning model by developing a food safety risk indicator system and integrating Support Vector Machine (SVM) technology. The model effectively handles small sample data, enables nonlinear predictions, and accurately identifies food safety risks. The results demonstrated that the proposed early warning model significantly enhanced the efficiency and reliability of food safety management, providing reliable early warning information for stakeholders across the food industry chain. Sheng et al. [15] proposed a food safety risk assessment model that combines the Analytic Hierarchy Process (AHP) with the Boosting-based XGBoost algorithm. Through empirical analysis of rice hazard factor detection data from 31 provinces, the study demonstrated that the model exhibited excellent performance in terms of smoothness and prediction accuracy, effectively assessing food safety risks. However, despite the successes of these methods, they still face limitations when handling complex, multidimensional data.

Artificial Neural Networks (ANNs) have gradually become a powerful data analysis tool for solving classification and regression prediction problems due to their ability to learn and model more complex data patterns [16]. Geng et al. [17] established an improved early warning method that combines the Cluster Hierarchical Analysis-Radial Basis Function (AHC-RBF) neural network with the Analytic Hierarchy Process (AHP) and Entropy Weight Method (EW). This model effectively predicts and manages food safety risks by integrating risk assessment with control measures. An application case using meat product detection data from a province in China validated the method’s effectiveness and feasibility in practical implementation. Niu et al. [18] used a safety risk assessment and early warning model for chemical contaminants in edible vegetable oils. The model integrates dietary exposure assessment and the Margin of Exposure (MOE) method and establishes an early warning system for food oil safety risks using the Analytic Hierarchy Process (AHP) and Backpropagation (BP) neural network. The study demonstrated that the BP neural network model used for predicting chemical hazard risks in edible vegetable oils offers good stability and accuracy, effectively providing risk management references for relevant authorities and enhancing food safety regulation. Geng et al. [19] proposed a food safety early warning model that combines the Deep Radial Basis Function (DRBF) neural network with the Analytic Hierarchy Process (AHP). The study demonstrated that the model effectively handles complex food safety detection data, showing strong predictive ability and high accuracy. However, artificial neural networks are prone to overfitting when handling complex data, exhibit slower convergence rates, and are susceptible to local optima during the training process [20].

In the realm of grain and oil food safety supervision, high-precision risk prediction is not merely a technical metric but a critical industry necessity for safeguarding public health and optimizing regulatory decision-making [21,22]. However, existing methods often fall short of meeting the rigorous standards required for real-world applications when processing complex detection data. While traditional models suffer from capacity constraints and standard neural networks struggle with stability on small datasets, the collective consequence is a lack of reliable risk prediction mechanisms [23]. To effectively address these limitations, the TabNet-BO model is proposed as a solution. It overcomes the limitations of standard neural networks by utilizing a sequential attention mechanism that explicitly addresses complex feature interactions, enabling the model to focus on critical risk indicators at each decision step. Furthermore, to alleviate the instability associated with small-sample training, the model adopts a sparse activation strategy. This mechanism emulates the interpretability and robustness of decision trees, thereby significantly mitigating the risk of overfitting [24,25]. Finally, to maximize prediction accuracy, Bayesian Optimization is integrated to efficiently traverse the complex hyperparameter space, avoiding the sub-optimality often resulting from manual tuning [26].

At the same time, traditional risk prediction models typically rely on centralized or decentralized independent databases for data storage and management. However, empirical studies have exposed critical vulnerabilities in such architectures concerning food safety. For instance, Hu et al. [27] and Peng and Wang [28] have demonstrated that centralized storage is prone to unauthorized tampering and severe information silos, thereby undermining data reliability. Similarly, Biswas et al. [29] highlighted that the inherent traceability limitations in conventional supply chains inevitably erode consumer confidence in product quality. Furthermore, these fragmented systems are often hampered by incomplete data linkage and incompatible storage protocols, resulting in data opacity and the potential loss of traceable information, which significantly restricts coverage. Under such architectures, the reliability and traceability of model predictions are severely compromised. Consequently, there is a pressing need to leverage advanced technologies within food quality and safety management to guarantee data integrity and trustworthiness throughout the grain and oil supply chain.

Blockchain technology, as an emerging information technology has attracted significant attention in both academia and industry since its introduction by Satoshi Nakamoto in 2008 to support the decentralized cryptocurrency Bitcoin. It has since developed into a foundational technological architecture with promising applications across various domains [30]. Blockchain is essentially a distributed ledger system, with key characteristics including decentralization, immutability, transparency, and traceability [31,32]. By leveraging these intrinsic attributes, blockchain effectively overcomes the structural limitations of conventional architectures, as highlighted by recent comparative studies that demonstrate its significant superiority over traditional centralized systems. Ruan et al. [33] and Khanna et al. [34] pointed out that conventional systems relying on centralized databases suffer from structural defects, specifically susceptibility to data tampering and ‘single point of failure’ risks, which lead to information asymmetry. In contrast, blockchain mitigates these vulnerabilities by establishing a tamper-resistant trust foundation via decentralized ledgers. In terms of traceability performance, Malik et al. [35] conducted a quantitative comparison, revealing that traditional systems are often limited to partial traceability with a coverage rate of only 40–50%, whereas blockchain-based distributed architectures achieve 100% end-to-end coverage. Furthermore, in domain-specific applications such as smart agriculture and meat production, Rehman et al. [36] and Kaliji et al. [37] demonstrated that decentralized consensus mechanisms effectively overcome the data opacity and origin forgery issues prevalent in conventional systems. Building on these comparative advantages, blockchain technology demonstrates significant potential for food quality and safety management. Its distributed ledger structure and immutability ensure data integrity and trustworthiness, while transparency and traceability enable efficient data sharing. Advanced encryption algorithms enhance data security, and smart contracts streamline and automate the data verification process [38,39,40]. Overall, these features significantly enhance the collaborative efficiency between institutions, thereby effectively ensuring the safety of grain and oil food.

To address issues such as unreliable data storage, low accuracy and credibility of prediction results, and lack of effective traceability in traditional grain and oil food quality safety management, this study proposes a grain and oil food quality safety risk prediction model that combines blockchain and deep learning. The model first uses Grey Relational Analysis (GRA) and a Bayesian-optimized Tabular Neural Network (TabNet-BO) for risk prediction, enabling efficient and accurate analysis of grain and oil food quality risks. Secondly, blockchain technology is utilized to record exceeding data and prediction results on the blockchain, ensuring the authenticity and traceability of the data. Finally, a storage optimization approach is introduced, where only exceeding data is uploaded to the blockchain, while non-exceeding data is encrypted and stored in a local database, effectively reducing the storage burden on the blockchain.

## 2. Materials and Methods

### 2.1. Blockchain

**Figure 1:**
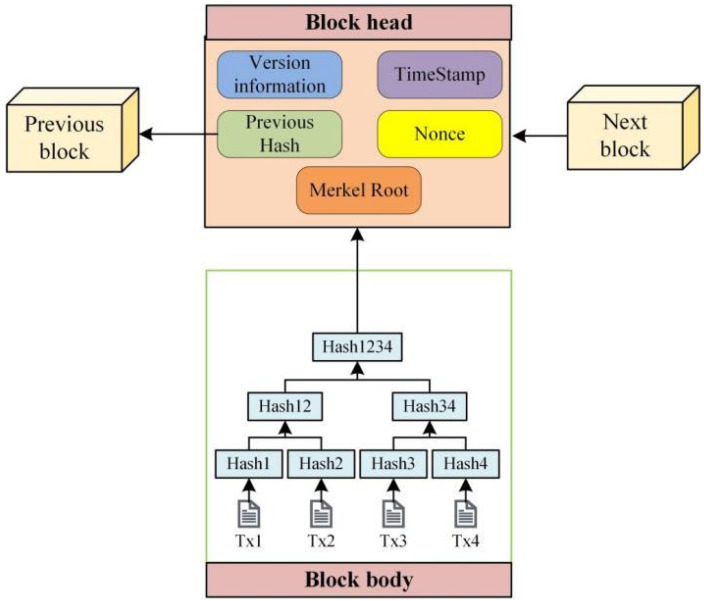
Block structure diagram.

Blockchain consists of a series of blocks connected in a specific order. Each block contains transaction data and is linked to the previous block through an encrypted hash, forming a chain structure [41]. Once a block is added to the chain, it cannot be deleted or modified. Each block is made up of two parts: the block header and the block body. The block header includes the hash of the previous block, a timestamp, and other metadata, ensuring that the blocks are linked in strict chronological order. The block body contains the number of transactions within the current block and all transaction records generated during the block’s creation. These records are processed using a Merkle tree hash, which produces a unique Merkle root that is stored in the block header [42,43], as shown in Figure 1.

Blockchain is essentially a decentralized database system with distributed storage characteristics [44]. This means that data is not stored on a single centralized server but is distributed across multiple nodes in the network, with each node maintaining a complete copy of the ledger. In contrast to traditional centralized databases, the decentralized architecture of blockchain obviates the need for a central authority, effectively eliminating the ‘single point of trust.’ This structural shift mitigates trust-related vulnerabilities and enhances the system’s resilience against unauthorized data tampering. Blockchain links each block using a hash algorithm, with each block containing the hash value of the previous block [45]. Any modification to recorded data would alter the hash values of that block and subsequent blocks, making tampering easily detectable by other nodes in the network, thereby enhancing data security and immutability [46]. The consensus mechanism of blockchain further enhances tamper resistance and data reliability. Each node executes complex algorithms and verifies the validity of transactions to ensure that all blocks comply with predefined rules, rejecting any invalid or tampered data.

### 2.2. SM2 Algorithm

The SM2 algorithm is a public-key encryption technology based on elliptic curve cryptography (ECC) developed by the State Cryptography Administration of China [47]. Its core functions include asymmetric operations for encryption and decryption, as well as generation and verification of digital signatures. The SM2 algorithm offers significant advantages in cryptographic technology due to high security, low storage requirements, and fast signature processing speed. Its encryption process is based on elliptic curves over a 256-bit prime field, with the specific elliptic curve equation being: $y^{2}=x^{3}+a_{x}+b$, where a and b are constants in the prime field that determine the shape of the elliptic curve. The signing process of the SM2 algorithm first obtains the hash value from plaintext m, then uses private key d_A_ to generate the signature value (R, S). This process first requires the generation of a key pair, where the private key is d_A_ and the public key is P_A_, $P_{A}=d_{A}G=\left(x_{A},y_{A}\right)$. The sender calculates the digest by hashing the plaintext and performs the signature calculation using the private key.

### 2.3. Smart Contract

A smart contract is an agreement that automatically executes predefined rules and triggers related operations when specific conditions are met [48]. It can complete designated tasks based on predefined rules without the intervention of intermediaries or third parties. Once deployed on a blockchain platform, a smart contract cannot be modified or deleted, ensuring the immutability and security of its content. This characteristic has led to the widespread application of smart contracts in fields such as finance, insurance, and real estate, which require a high degree of trust. Additionally, the execution process of smart contracts is completely transparent, allowing all participants to view the contract’s execution status in real time and verify the specific details of each transaction on the blockchain [49]. The decentralized nature of blockchain further ensures the fairness and accuracy of contract execution results. Smart contract operations are validated through the blockchain’s consensus mechanism, which not only ensures the correctness of the operations but also enhances the credibility of transactions.

### 2.4. GRA-TabNet-BO Risk Prediction Model

#### 2.4.1. Architectural Framework of the GRA-TabNet-BO Model

**Figure 2:**
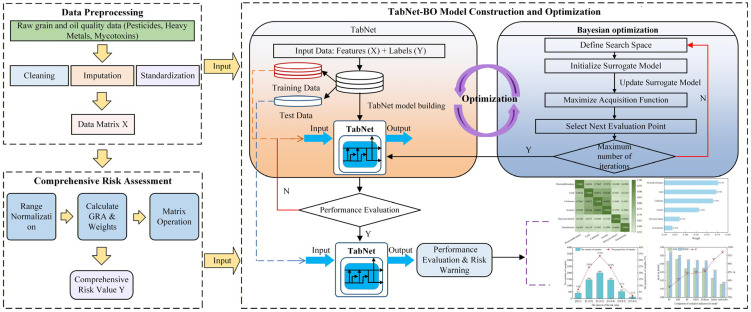
The framework of GRA-TabNet-BO.

In order to improve the efficiency of predicting the quality and safety risks of grain and oil food and to overcome the limitations of traditional models, this study proposes a new prediction model. The model combines gray relational analysis (GRA) with the TabNet-BO model and, through data preprocessing, risk assessment, and model training steps, effectively improves the accuracy and reliability of grain and oil food safety risk prediction. The overall process is shown in Figure 2.

Step 1: Data Preprocessing. Raw detection datasets, encompassing critical quality indicators such as pesticide residues, heavy metal content, and mycotoxin levels, undergo rigorous cleansing and preprocessing. This phase involves the elimination of redundant features and the imputation of missing values utilizing mean or interpolation strategies. Subsequently, data formats are standardized to ensure integrity and consistency, generating a normalized data matrix optimized for analytical modeling.

Step 2: Comprehensive Risk Quantitative Evaluation Based on Grey Relational Analysis. Grey Relational Analysis (GRA) is employed to quantify the aggregate risk value of grain and oil samples. Initially, range normalization is applied to standardize detection data, thereby neutralizing dimensional disparities across different measurement units. The grey relational degree between each indicator and the target risk value is then computed to assess the strength of inter-feature correlations, assigning weights accordingly. Finally, a comprehensive risk value for each sample is derived through matrix operations that integrate the standardized data with the calculated weight vectors.

Step 3: Construction and Optimization of the TabNet-BO Model. The TabNet architecture is deployed to execute automated feature selection and risk prediction, leveraging self-attention mechanisms combined with decision-tree structures to extract salient features effectively. To maximize predictive performance, Bayesian Optimization (BO) is integrated to autonomously fine-tune the model’s hyperparameters. The process concludes with a comprehensive performance evaluation to validate the model’s effectiveness, accuracy, and robustness, ensuring its stability for practical applications.

#### 2.4.2. The Grey Relational Analysis

Grey Relational Analysis (GRA) is an important component of grey system theory, specifically used for multi-criteria decision-making and comprehensive evaluation. Its core is to quantify the geometric similarity between the reference sequence and multiple comparison sequences, thereby assessing the degree of correlation between the sequences [50,51]. Compared with traditional parametric approaches (e.g., PCA), GRA demonstrates distinct methodological superiority for large-scale risk assessment. First, as a non-parametric technique grounded in geometric similarity, GRA circumvents strict distributional assumptions (e.g., Gaussian), thereby exhibiting high adaptability to the irregular data distributions characteristic of real-world scenarios. Moreover, applying GRA to this large-scale dataset bolsters statistical stability; the aggregation of relational degrees effectively attenuates random noise, ensuring that the derived weights reflect global risk characteristics rather than local biases. Second, within the proposed framework, GRA serves as a Multi-Criteria Decision Making (MCDM) instrument to quantify the ‘Comprehensive Risk Value’ as the target label. Unlike simple linear correlation metrics, GRA evaluates geometric curve similarity to capture intrinsic trend-based relationships. This capability yields a deterministic and interpretable metric, establishing an objective ‘Ground Truth’ for subsequent risk analysis.

To comprehensively assess the risk level of grain and oil food, this study employs the Grey Relational Analysis (GRA) method. By performing a weighted fusion of multiple detection indicators, the comprehensive risk value for each sample is calculated. The specific process is as follows:

(1) Data Normalization. The detection data of grain and oil food is standardized. Let the detection data matrix be denoted as X, where *X_ij_* represents the detection value of the *i*-th indicator in the *j*-th sample. To eliminate the dimensional influence between different indicators, the range normalization method is applied to normalize each indicator. The standardization formula is as follows:

(1) $$Z_{ij}=\frac{X_{ij}-\min(X_{i})}{\max(X_{i})-\min(X_{i})}$$

where min(*X_i_*) and max(*X_i_*) are the minimum and maximum values of the *i*-th indicator, and *Z_ij_* is the normalized data.

(2) Calculation of the grey relational coefficient of the sample. When calculating the grey relational coefficient for sample k, the reference sequence is the target risk value sequence *z_1_(k)*. The grey relational coefficient *γ_1k_* for sample k and the grey relational coefficient *γ_ik_* for other samples are calculated as follows:

(2) $$\gamma_{ik}=\frac{\underset{j}{\min}\left|z_{j}(k)-Z_{ij}\right|+\rho \underset{j}{\max}\left|z_{j}(k)-Z_{ij}\right|}{\left|z_{1}(k)-Z_{ij}\right|+\rho \underset{j}{\max}\left|z_{j}(k)-Z_{ij}\right|}$$

where *z_1_(k)* is the target risk sequence, *Z_ij_* is the standardized data of sample *k*, and *ρ* is the distinguishability factor, used to adjust the differences between the correlation coefficients (ρ ∈ (0, 1)); when ρ is smaller, the differences between the correlation coefficients are larger, and the distinguishability is stronger. It is usually set to ρ = 0.5.

(3) Calculate the correlation coefficients between the sequences. The grey relational degree between all indicators and the target sequence is calculated, yielding the correlation coefficient *γ_ik_*, for each sample in relation to the target sequence. The grey relational degree between the sequences is then determined using the following formula:

(3) $$\gamma_{ij}=\frac{\underset{k}{\min}\left|r_{k}-Z_{ik}\right|+\rho \underset{k}{\max}\left|r_{k}-Z_{ik}\right|}{\left|r_{i}-Z_{ij}\right|+\rho \underset{k}{\max}\left|r_{k}-Z_{ik}\right|}$$

where *r_k_* is the target risk value, *Z_ik_* is the standardized data of the *i*-th indicator in the k-th sample, and *γ_ij_* represents the correlation degree between the *i*-th indicator and the j-th sample.

(4) Establish the correlation coefficient matrix. The correlation coefficients *γ_ij_* of all samples are combined to form a correlation coefficient matrix Γ, as follows:

(4) $$\Gamma =\left(\begin{array}{ccc} \gamma_{11} & \ldots & \gamma_{1n}\\ \vdots & \ddots & \vdots \\ \gamma_{m1} & \ldots & \gamma_{mn} \end{array}\right)$$

where the elements *γ_ij_* of the matrix Γ represent the grey relational degree between the *i*-th indicator and the *j*-th sample. This matrix reflects the relationship between all samples and the reference sequence.

(5) Calculate the risk contribution weights. Based on the grey relational analysis, the weight *ω_i_* is derived to quantify the relative contribution of each indicator to the composite risk definition. The final weight is obtained by normalizing the correlation degree of each indicator with the target sequence. This ensures that the ‘Comprehensive Risk Value’ reflects a balanced integration of hazard factors based on geometric proximity, as shown in the following formula:

(5) $$\omega_{i}=\frac{\sum_{k=1}^{n}\gamma_{ik}}{\sum_{i=1}^{m}\sum_{k=1}^{n}\gamma_{ik}}$$

where *ω_i_* is the weight of the *i*-th indicator, reflecting the degree of contribution of the indicator to the sample risk.

(6) Calculate the sample risk value. By combining the weights of each indicator with the standardized data of the sample, the comprehensive risk value *R_k_* of each sample can be calculated. The specific calculation formula is as follows:

(6) $$R_{k}=\sum_{i=1}^{m}\omega_{i}\cdot Z_{ik}$$

where *R_k_* is the comprehensive risk value of the k-th sample, *Z_ik_* is the standardized data of the *i*-th indicator in the *k*-th sample, and *ω_i_* is the weight of the *i*-th indicator.

#### 2.4.3. TabNet Model Based on Bayesian Optimization

##### TabNet Model

TabNet is an innovative deep learning model specifically designed for processing tabular data. It adopts a tree-like structure and assigns coefficients to determine the importance of these specific features in the decision-making process, thereby promoting the effective combination of features. Through a sparse, instance-wise feature selection mechanism, TabNet learns to identify and prioritize the most relevant risk indicators (e.g., distinguishing between heavy metal contamination and mycotoxin levels) at each step of the decision process. This approach allows the model to dynamically adjust the weight of each hazard factor, enhancing the model’s representational capabilities and improving efficiency and accuracy.

**Figure 3:**
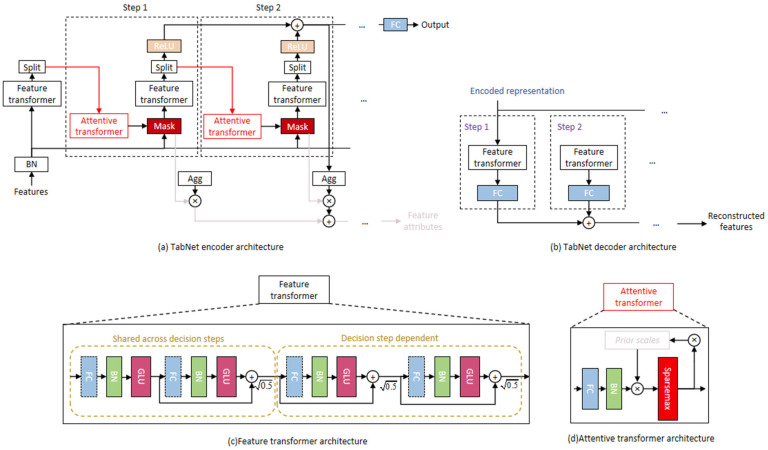
TabNet Model Architecture.

Additionally, TabNet employs a multi-step continuous architecture, processing D-dimensional feature vectors over *n* steps and feeding them into the feature transformation module. This module consists of a fully connected layer, a batch normalization layer, and an activation function based on gated linear units (GLU), where the GLU captures nonlinear feature relationships through gating operations. Residual normalization connections help maintain network variance and enhance training stability. The feature transformer is linked to a masking module to ensure reliable selection of relevant safety risk features at each stage, adapting to the processing of complex tabular data. This feature selection mechanism enables TabNet to train more efficiently when handling data with a large number of features, thereby reducing computational resource consumption. Furthermore, the multi-stage structure of TabNet allows flexible adjustment of decision strategies at each step, continuously optimizing performance, as shown in Figure 3.

##### Bayesian Optimization for Hyperparameter Tuning

Bayesian Optimization (BO) is a global optimization method based on probabilistic models, widely applied to address challenges in optimization tasks characterized by high dimensionality, high computational cost, and the absence of analytical solutions. In particular, Bayesian Optimization is considered a highly effective technique for hyperparameter tuning in deep learning models. It constructs a surrogate model (typically a Gaussian process) to predict the potential behavior of the objective function, thereby intelligently guiding the search process to find the optimal solution with the fewest possible experiments. This makes Bayesian Optimization particularly suitable for complex models with long training times and vast hyperparameter spaces.

In practical applications, hyperparameter tuning is a critical step in improving model performance. Traditional hyperparameter tuning methods, such as grid search and random search, require traversing all or part of the hyperparameter space, which can result in significant computational overhead and time costs in high-dimensional, complex optimization problems. In Bayesian optimization, a surrogate model (typically a Gaussian process, GP) is established to approximate the objective function and guide the search process. The core idea of Bayesian optimization is to use the predictions of the surrogate model to select the hyperparameter combination most likely to improve the objective function value in the next step, thereby efficiently finding the optimal solution with a limited number of experiments. Therefore, using Bayesian optimization to replace traditional methods not only significantly improves tuning efficiency but also better addresses hyperparameter optimization problems when resources are limited.

### 2.5. Risk Prediction Framework Based on Blockchain and Deep Learning

**Figure 4:**
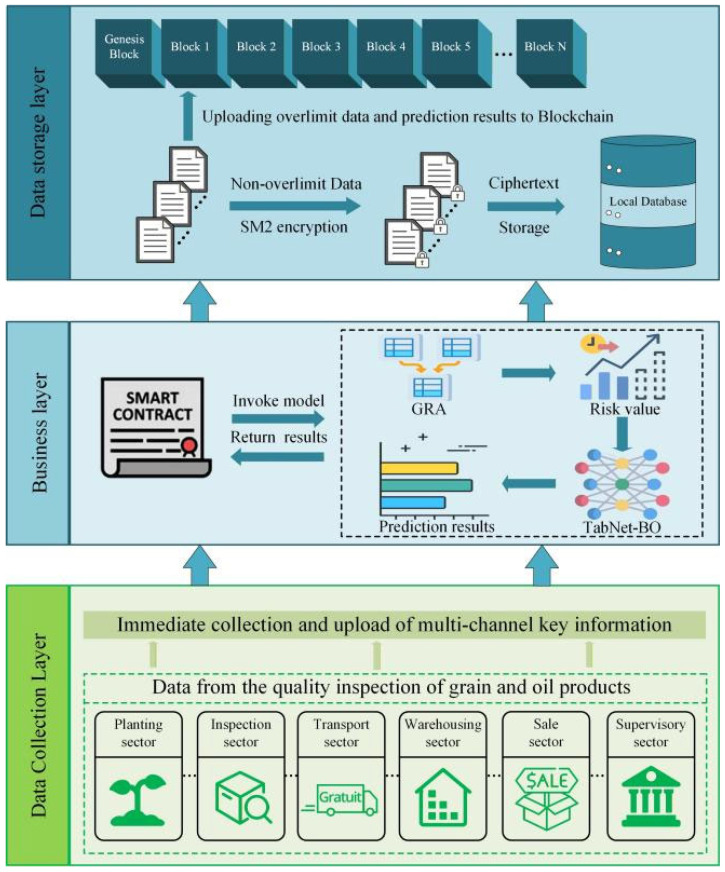
Risk prediction framework integrating blockchain and deep learning.

The quality and safety of grain and oil food directly impact public health and the sustainable development of society. Effective risk prediction not only enables the early identification of potential safety hazards but also provides critical data support for the formulation of scientific regulatory measures. While existing risk prediction models are effective in certain aspects, they often face issues of insufficient data reliability and lack of traceability. To address these issues, this study proposes a risk prediction framework based on blockchain and deep learning, combining deep learning with blockchain technology. Through multi-level processing and storage, this framework ensures the reliability and traceability of source data and prediction results, as shown in Figure 4.

The framework is divided into three layers: the data collection layer, the business layer, and the data storage layer. Each layer plays a distinct and important role in the entire process, working together to ensure comprehensive monitoring, early warning, and traceability of grain and oil food quality.

(1) Data Collection Layer: The data collection layer is the foundational layer of the framework, responsible for collecting raw data from various stages of grain and oil food production and testing. By establishing diversified data collection mechanisms, it ensures the comprehensiveness and completeness of the data. This data includes quality testing values (such as pesticide residues, mycotoxins, heavy metal contamination, etc.) and key information such as production batches, enabling comprehensive monitoring and traceability of grain and oil food quality. The key task of the data collection layer is to ensure the reliability and integrity of data sources, build a stable data collection network, prevent data omission or tampering, and provide robust data support for subsequent quality assessment, risk prediction, and regulatory oversight.
(2) Business Layer: The business layer is the core component of the framework, responsible for screening data exceeding quality and safety standards for grain and oil food products and for predicting risks. This layer integrates deep learning models with smart contracts to ensure data credibility and traceability. First, smart contracts automatically screen uploaded data based on predefined quality standards. Because smart contracts execute without human intervention, they automatically identify data exceeding standards. This automated screening process reduces the time and error rates associated with manual review, ensuring efficient and consistent data processing. Next, a pre-trained deep learning model interface is invoked for risk prediction. This model combines gray relational analysis (GRA) with Bayesian optimization of tabular neural networks (TabNet-BO) to provide accurate, granular predictions, effectively identifying potential quality risks. Finally, exceeding data (such as product information and test values) and prediction results (such as predicted values and risk levels) are uploaded to the blockchain. The blockchain not only ensures immutability and decentralization but also enhances data transparency and traceability—no party can alter the uploaded data, significantly boosting security and credibility.
(3) Data Storage Layer: The data storage layer is responsible for data storage and management. To address the storage limitations of blockchain, this layer employs a two-tier storage strategy. Exceeding data (such as quality inspection values or prediction results) filtered by the smart contract will be uploaded to the blockchain. Due to the high cost and limitations of blockchain storage, only data critical to quality and safety will be uploaded. Non-exceeding data is stored in a local database, where it is protected using the SM2 encryption storage mechanism. The SM2 algorithm, as a public-key cryptography scheme, ensures that data remains unaltered and secure during storage. By uploading only exceeding data to the blockchain, unnecessary data waste is avoided, optimizing storage efficiency. Additionally, the combination of a local database and blockchain enables the system to balance data security while effectively distributing storage pressure, thereby enhancing overall performance.

## 3. Results

### 3.1. System Efficiency Evaluation Under Tiered Storage Strategy

The computer hardware configuration used in the study is as follows: CPU, Intel Core i5-12490F 3.00 GHz; RAM, 16 GB; and hard disk capacity, 1 TB. The host operating system is Windows 10, within which the blockchain environment was implemented by deploying Hyperledger Fabric 1.2.0 on an Ubuntu 16.04 virtual machine managed by VMware Workstation. The detailed experimental environment configurations are listed in Table 1.

To realistically simulate the grain and oil supply chain environment, the experimental network topology is configured with five distinct Organizations (Orgs), each maintaining a dedicated Peer Node. In this topology, the Regulatory Organization (representing the Market Supervision Administration) is responsible for block generation and network governance. Two independent Inspection Organizations operate Endorsing Peers, verifying ‘exceeding data’ through digital signatures enforced by a strict endorsement policy. Meanwhile, Production Organizations (representing grain depots and processing enterprises) function as Committing Peers, responsible for uploading basic traceability information and synchronizing the ledger. Each organization maintains an independent Membership Service Provider (MSP) and implements role separation between administrators and ordinary users via its own Certificate Authority (CA) to enforce least privilege management. This distributed architecture, characterized by multi-party checks and balances, effectively circumvents single-point control risks and establishes data sovereignty and isolation.

For efficient data processing and system reliability, the Kafka consensus mechanism was adopted. Given the specific requirements of the grain and oil supply chain—which involves high-frequency data uploads from IoT devices and multiple participants—the system prioritizes high throughput and low latency over the absolute decentralization found in public chains. Kafka provides Crash Fault Tolerance (CFT), making it ideal for this permissioned consortium environment where nodes (such as government regulators and certified laboratories) are known, semi-trusted entities. Unlike Byzantine Fault Tolerance (BFT) mechanisms, which are computationally expensive, Kafka effectively handles node failures while maintaining the high ordering performance required for real-time traceability in Hyperledger Fabric v1.2.0.

This study adopted a two-tier storage strategy, specifically uploading only exceeding data to the blockchain, while non-exceeding data were stored locally in an encrypted database. Through this strategy, the system optimizes storage efficiency while ensuring the security and traceability of critical data. First, the validity and compliance of the detection data are strictly verified, and precise analyses are conducted on the content of various harmful substances such as pesticide residues and heavy metal contamination. Once the detection values exceed safety standards, the system marks the data as “exceeding data” and uploads it to the blockchain system for subsequent traceability and regulation.

**Figure 5:**
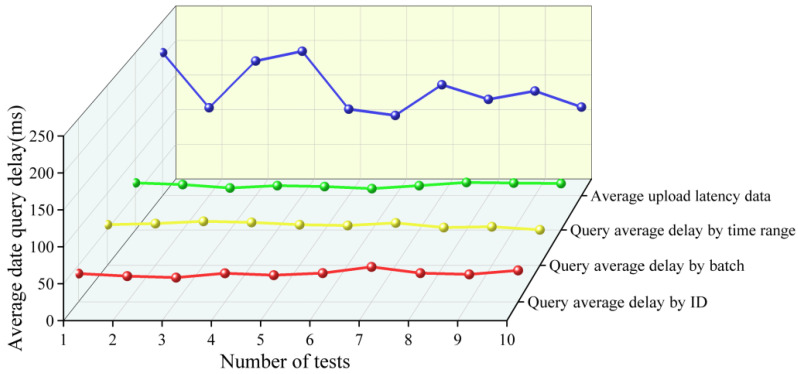
Average data upload and query latency.

During the upload process, blockchain nodes first initiate a transaction request via smart contracts. Next, ordering nodes receive the transaction data and generate a block. Finally, the generated block is broadcast to other peers in the network for synchronization. The method used for data upload underwent 10 rounds of testing, with 20 data uploads per round. The results showed an average upload latency of 0.154 s, meeting the requirements for data upload. Since both grain and oil food quality testing data and predictive data are stored on the blockchain, this study provides three query methods to meet different data retrieval requirements: ID query, batch query, and time-range query. The data query methods were tested over 10 rounds, with 20 data queries conducted in each round. The results showed average latencies of 38.9 ms for ID queries, 56.1 ms for batch queries, and 63.3 ms for time-range queries, meeting the requirements for data retrieval, as shown in Figure 5.

**Figure 6:**
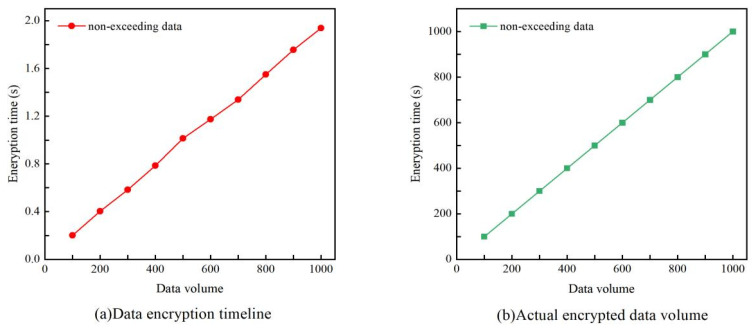
Data encryption performance at different data volumes.

Non-exceeding data will be encrypted using the SM2 algorithm and stored in the local database. To evaluate the efficiency and reliability of the encryption process, we tested the time consumption and success rate of encryption for different data volumes. First, we set up 200, 400, 600, 800, and 1000 data records and encrypted them using the SM2 algorithm. The encryption time for each record was recorded, and the average encryption time was calculated. The experimental results indicate that as data volume increases, encryption time exhibits linear growth, demonstrating that the SM2 algorithm maintains stable performance and good scalability when processing different data volumes. In terms of encryption success rate, all data in the experiment was successfully encrypted, indicating that the SM2 algorithm has high reliability. This means that under the given experimental conditions, the SM2 encryption algorithm can stably complete encryption tasks and ensure data security, as shown in Figure 6.

### 3.2. Performance Evaluation of the Risk Prediction Model

#### 3.2.1. Risk Dataset Analysis and Model Configuration

**Figure 7:**
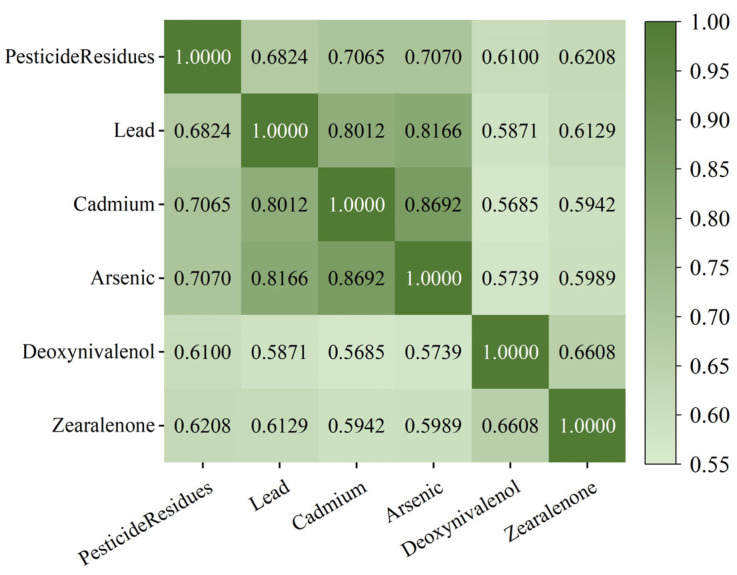
Correlation Matrix Heatmap of Risk Indicators.

**Figure 8:**
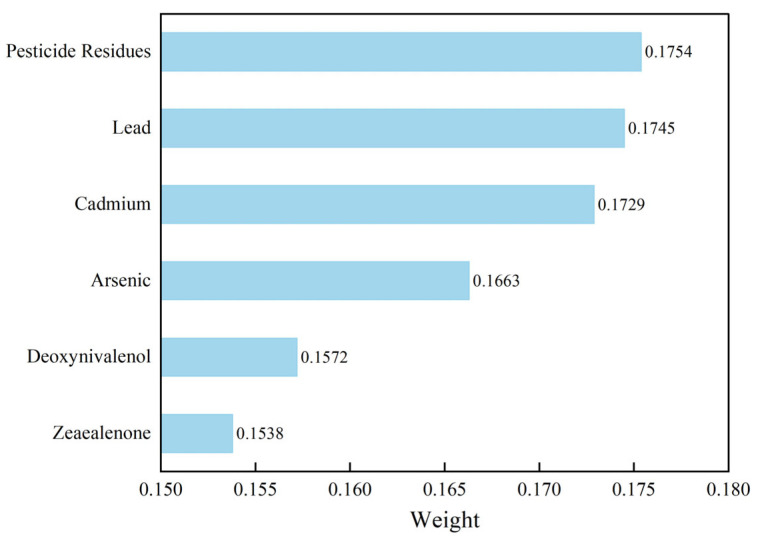
Weight Distribution of Risk Indicators.

This study utilizes 6785 sets of data provided by a grain and oil food quality testing institution in Henan Province, China. The data collection spans from 2018 to 2023, covering samples from multiple regions within the province. The dataset specifically focuses on wheat, a major staple grain. Each sample contains six key attributes: pesticide residues, lead, cadmium, arsenic, Deoxynivalenol (DON), and Zearalenone (ZEN). The selected samples were sourced from diverse storage environments and varying production batches over this five-year period, encompassing broad environmental conditions. Given that Henan is the largest wheat-producing province in China, this dataset is highly representative of typical wheat safety risks. This diversity supports the model’s potential generalizability to other major wheat-producing regions with similar agricultural and climatic characteristics. Following data collection, the GRA method was used to perform correlation analysis on the sample data and calculate the weights of each evaluation indicator. The content data of various harmful substances in the grain and oil food samples were integrated with the corresponding indicator weights to derive the comprehensive risk value for each sample. By calculating the correlation coefficients between each evaluation indicator, their degree of association was assessed; the larger the correlation coefficient, the stronger the association between the two indicators. The correlation of the evaluation indicators and their heatmap matrix are shown in Figure 7. Based on the correlation coefficient matrix, the weights for each risk assessment indicator were further calculated, with the results shown in Figure 8. Finally, these weights were used to calculate the comprehensive risk value for each grain and oil food sample, with the sample data serving as input for the early warning model and the comprehensive risk value as the expected output for training the risk prediction model.

**Figure 9:**
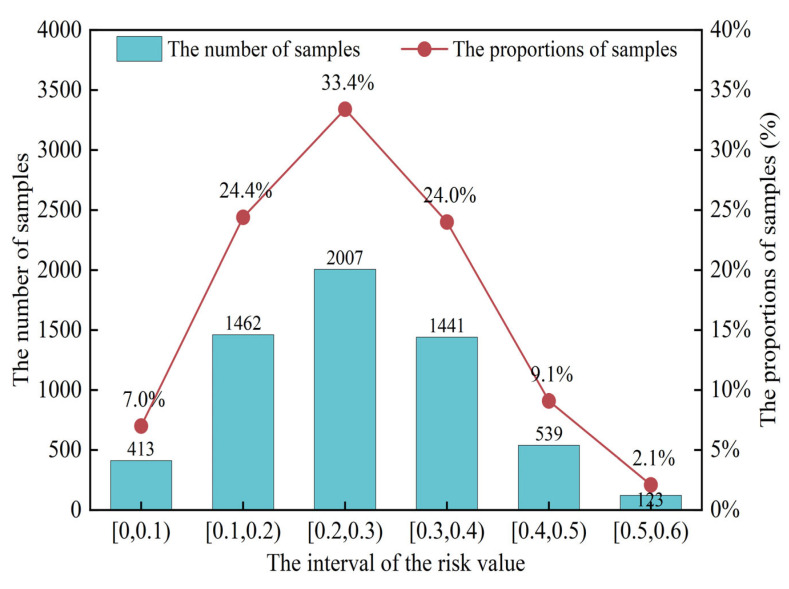
Comprehensive Risk Value Distribution Chart.

The weights assigned to each risk factor through methods such as grey relational analysis (GRA) enable the weighted integration of multiple testing indicators for each grain and oil food sample, thereby calculating a comprehensive risk value. Here, the sample data serves as input for the risk prediction model, while the comprehensive risk value functions as the expected output for training the risk prediction model. As shown in Figure 9, the risk values of grain and oil food samples exhibit distinct distribution patterns across different intervals: The [0.2, 0.3) interval contains the highest number of samples (2007), accounting for 33.4%, indicating this range as the “concentrated distribution zone” for grain and oil food risks; The [0.1, 0.2) interval contains 1462 samples (24.4%), representing the second-highest risk interval; The [0.3, 0.4) interval has 1441 samples (24.0%); The [0.0, 0.1) and [0.4, 0.5) intervals have relatively fewer samples, with 413 (7.0%) and 530 (9.1%) respectively; The [0.5, 0.6) interval has the fewest samples, only 211 (3.5%).

To effectively capture the characteristics of this data distribution and achieve optimal convergence, training hyperparameters were specifically optimized for each model architecture, as presented in Table 2. Deep learning baselines (TabNet-Default, BP) employed extended epochs (500 and 400) with dynamic schedulers (e.g., ReduceLROnPlateau) to ensure stability. The proposed TabNet-BO utilized Bayesian Optimization to fix an optimal learning rate of 0.0031; while this increased training time to 1046 s, it was critical for capturing complex non-linear interactions. In contrast, ensemble models (XGBoost, GBDT) relied on high estimator counts (800) to minimize variance. Although these models achieved high computational efficiency (19–25 s), their simpler structures limit their ability to capture high-dimensional dependencies compared to deep learning architectures.

Specifically for the proposed architecture, Bayesian Optimization guided the search process to balance predictive accuracy with stability. The optimization traversed the architectural space of [8, 128] for widths (N_d_, N_a_) and [3, 10] for decision steps (N_step_), ultimately converging to an optimal configuration of N_d_ = 56, N_a_ = 8, and N_step_ = 7. Simultaneously, the training parameters were fine-tuned to ensure robust convergence, settling at a learning rate (η) of 0.0031 and relaxation parameter (γ) of 1.43, as detailed in Table 3.

#### 3.2.2. Model Performance Evaluation

To evaluate the predictive performance of the model, this study conducted comparative experiments on the same dataset between the TabNet-BO model and six other predictive models. The selected baselines include traditional machine learning models (RF, GBDT, XGBoost, and RBF (SVR)) and deep learning models (BP and TabNet). Among the traditional methods, RF aggregates predictions from multiple decision trees, while GBDT and XGBoost optimize performance through residual correction and regularized parallel computing, respectively. RBF (SVR) captures non-linearities by mapping inputs to high-dimensional spaces. In the deep learning domain, BP utilizes error backpropagation, whereas TabNet employs attention mechanisms for automated feature selection in tabular data.

**Figure 10:**
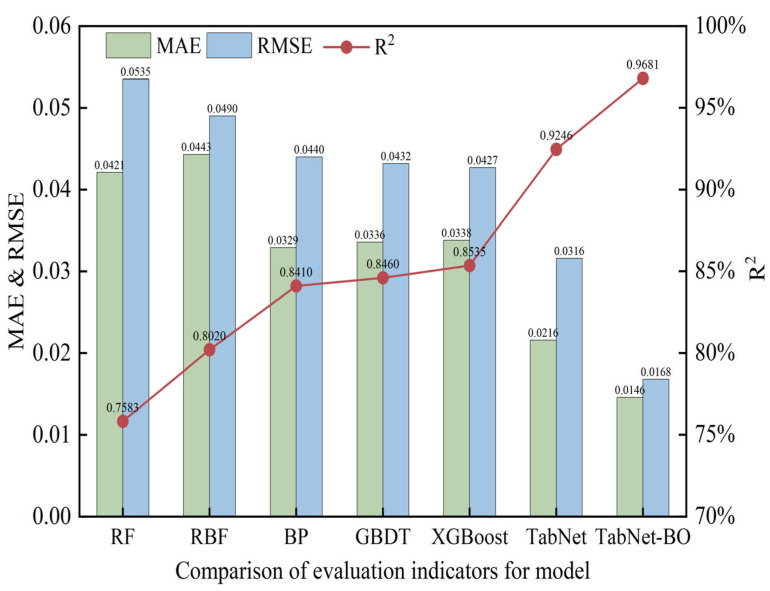
Comparison of Model Evaluation Metrics.

The evaluation metrics for generalization performance quantitatively measure the generalization ability of risk prediction models. On the test set, model performance can be evaluated using three metrics: root mean square error (RMSE), mean absolute error (MAE), and coefficient of determination (R^2^). For RMSE and MAE, smaller values indicate stronger risk prediction capabilities of the model. For R^2^, the closer the indicator value is to 1, the higher the model’s fitting accuracy to the fluctuations in the actual values in the prediction. Relative metrics such as MAPE were excluded to avoid numerical instability caused by the near-zero risk values observed in Figure 9 (e.g., the [0.0, 0.1) interval), which would otherwise result in artificially inflated error rates. The results are shown in Table 4 and Figure 10.

**Figure 11:**
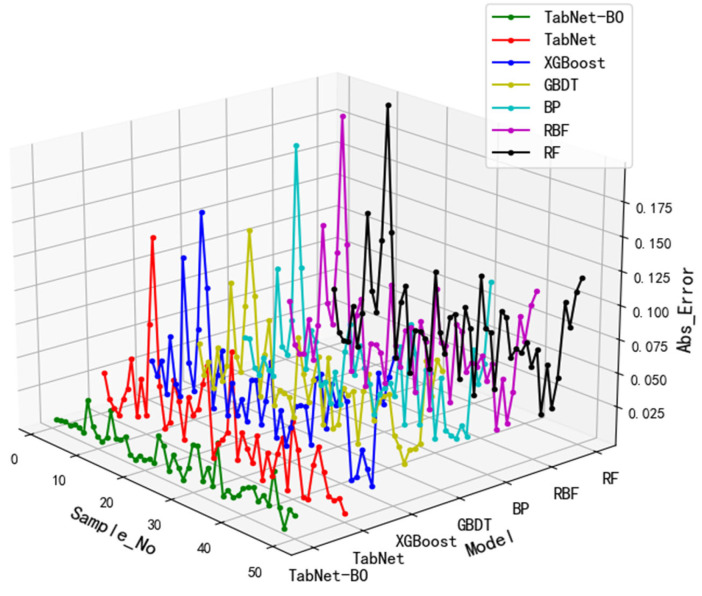
Absolute Prediction Error Curves of the Seven Models.

As shown in Table 4, the model based on TabNet-BO achieved the lowest values for both MAE (0.0146) and RMSE (0.0168) among the seven models, with an R^2^ value of 0.9681, the highest value closest to 1, indicating that this model demonstrates higher accuracy in predicting sample risk values. The absolute prediction error curves for the seven models are shown in Figure 11. Absolute error is the absolute value of the difference between the model’s predicted value and the true value. As observed in Figure 11, the error curve of the TabNet-BO model exhibits the smallest fluctuation amplitude, demonstrating high stability.

**Figure 12:**
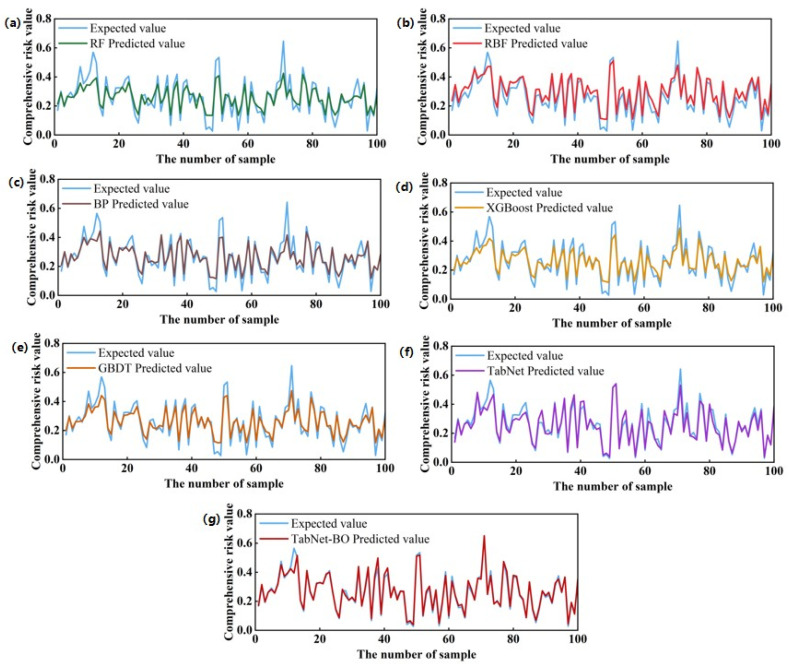
Comparison of actual values versus predicted values for the first 100 samples in the model: (**a**) RF, (**b**) RBF, (**c**) BP, (**d**) XGBoost, (**e**) GBDT, (**f**) TabNet, (**g**) TabNet-BO.

Figure 12 visually compares the prediction accuracy of seven models against actual composite risk values for the first 100 samples, further validating the differences in generalization performance when handling nonlinear risk data. Observation reveals that the TabNet-BO model exhibits optimal fitting capability, with its prediction curve demonstrating high consistency and near-perfect alignment with the true value curve. This indicates that the model not only accurately captures overall risk trends but also maintains extremely low deviation when confronting local extreme fluctuations. In contrast, other models exhibit varying degrees of lag and error: the unoptimized TabNet and ensemble models like XGBoost can fit the general trend but show noticeable residuals at peaks and troughs; while traditional models like RBF and RF exhibit significant deviation zones between their prediction curves and the actual curve, indicating substantial prediction errors. This visualization corroborates the quantitative evaluation metrics in Table 2 (R^2^ as high as 0.9681), fully confirming that incorporating Bayesian optimization effectively resolves model underfitting on complex samples, significantly enhancing the accuracy and robustness of food safety risk prediction for grain and oil food.

To rigorously validate the performance superiority of the proposed TabNet-BO framework, a Paired T-test was conducted to compare the prediction residuals of the proposed model against all baseline models on the identical test set. The null hypothesis (H_0_) posits that there is no significant difference in the mean absolute errors between the paired models. Table 5 summarizes the results. The positive T-statistics across all comparisons indicate that the prediction errors of the baseline models are consistently and significantly higher than those of TabNet-BO. The *p*-values for all pairs are well below the significance level of 0.05 (ranging from 10^−8^ to 10^−16^), leading to the strong rejection of the null hypothesis. This confirms that the TabNet-BO model achieves a statistically significant improvement in prediction accuracy over all comparative methods, including Gradient Boosting (GBDT, XGBoost), Random Forest (RF), RBF (SVR) and Deep Learning (TabNet, BP) baselines.

Crucially, this statistical robustness constitutes a functional prerequisite for the system’s automated execution protocols. As the smart contract executes the immutable recording of both the predicted risk values and the associated traceability data, the model’s demonstrated precision is pivotal in guaranteeing the validity of the recorded anomalies. This ensures that computationally expensive on-chain storage is allocated exclusively to genuine high-risk anomalies, thereby upholding the operational efficacy and cost-efficiency of the tiered storage strategy.

#### 3.2.3. Model Interpretability and Efficiency Analysis

Although the TabNet-BO model demonstrates superior predictive accuracy, its practical adoption in regulatory frameworks necessitates a validation of its decision logic against established agronomic principles. Feature mask analysis validates a ‘Dual-Risk Mechanism’ driven by Pesticide Residues and Deoxynivalenol (DON), aligning with the agronomic distinction between anthropogenic management errors and environmental climatic risks. However, in off-chain prediction, this inference logic remains an unverifiable ‘black box’ susceptible to data manipulation and lacking audit trails. The proposed on-chain architecture addresses this by leveraging smart contracts and consensus mechanisms to immutably record prediction results, traceability records, and decision information including feature weights and inference paths directly onto the distributed ledger, ensuring end-to-end data integrity from production detection to risk prediction. This transformation of opaque algorithmic outputs into transparent, tamper-proof evidence provides regulators with a solid trust anchor.

In parallel with interpretability, a critical evaluation of the experimental results highlights a necessary trade-off between computational cost and predictive precision. As indicated in the experimental setup (Table 2), the proposed TabNet-BO model incurs a considerably higher training latency compared to traditional machine learning baselines such as Random Forest. However, this increased temporal cost is primarily attributed to the iterative exploration process during the initial Bayesian hyperparameter search. It represents a one-time offline initialization cost aimed at identifying the global optimum. In practical deployment phases, where the hyperparameters are already fixed (as listed in Table 3), the model operates without this search overhead, meaning the recurring computational cost is substantially lower. This computational investment yields substantial returns in predictive accuracy. The optimized model achieved an R^2^ of 0.9681, significantly outperforming the computationally cheaper ensemble models (R^2^ ranging from 0.7583 to 0.8535). In the specific context of grain and oil food supervision, where high predictive precision enables the targeted allocation of limited regulatory resources, the priority is unequivocally placed on model sensitivity rather than training speed. Therefore, the one-time cost of extended training is justified by the model’s superior capability to capture complex non-linear risk interactions that simpler, faster models fail to resolve.

## 4. Conclusions

This study proposes a blockchain-based risk prediction model for grain and oil food quality and safety. First, efficient risk prediction is achieved by introducing the Grey Relational Analysis (GRA) and Bayesian Optimization-based Tabular Neural Network (TabNet-BO) model, thereby improving the accuracy of grain and oil food quality risk prediction. The experimental results show that this model demonstrates significant performance in grain and oil food quality risk prediction, with an accuracy rate exceeding 96% and an MAE of 0.0146 and RMSE of 0.0168, confirming the model’s advantages in handling complex data patterns and diverse risks. Next, to ensure the credibility and traceability of the prediction results, blockchain technology is used to record exceeding data and prediction results, ensuring the authenticity and transparency of the data. Data interaction and verification are completed through smart contracts, further enhancing data security. Finally, the proposed storage optimization method significantly reduces the storage pressure on the blockchain by only uploading exceeding data to the blockchain, while encrypting and storing non-exceeding data in a local database, thus improving the system’s storage efficiency. The experimental results indicate that the model performs well in risk prediction, data uploading, and querying. Collectively, these architectural safeguards—specifically the tiered storage strategy coupled with Kafka-based ordering—ensure robust viability in geographically dispersed deployments, keeping network latency well within the operational tolerances of grain and oil supply chains.

As application scenarios evolve into complex contexts involving large-scale, multi-source heterogeneous data, guaranteeing input data quality and overcoming legacy integration barriers become as critical as safeguarding the synergy between the prediction model and blockchain while upholding the latter’s distributed immutability, traceability and storage efficiency and addressing cross-organizational privacy compliance. Future research will focus on integrating Federated Learning to resolve privacy concerns without compromising blockchain-enabled data credibility, leveraging lightweight edge computing to support coordinated operation of the prediction model and blockchain on resource-constrained legacy devices, and extending this integrated framework to other high-risk domains such as meat and dairy cold-chain logistics.

## Figures and Tables

**Table 1:** Experimental environment.

Environment	Description
Development Platform	IntelliJ IDEA 2019.3.3 × 64; MySQL8.0
Operating System	Windows 10; Ubuntu 16.04
Blockchain Module	Hyperledger Fabric v1.2.0; nodejs v8.10.0; go v1.12
Languages	Python 3.8; Go; Node.js

**Table 2:** Detailed experimental setup and training configurations for all models.

Model	Type	Epochs/Estimators	Learning Rate (Init)	Scheduler	Training Time (s)
TabNet-BO (Proposed)	DL	500	0.0031	ReduceLROnPlateau	1046
TabNet (Default)	DL	500	0.005	ReduceLROnPlateau	415
BP	DL	400	0.001	StepLR	163
XGBoost	ML	800	N/A	N/A	25
GBDT	ML	800	N/A	N/A	19
Random Forest (RF)	ML	300	N/A	N/A	7
RBF(SVR)	ML	N/A	N/A	N/A	4

**Table 3:** Hyperparameter search ranges and optimal values for the TabNet-BO model.

Hyperparameter	Symbol	Search Range	Optimal Value
Decision prediction width	N_d_	[8, 128]	56
Attention prediction width	N_a_	[8, 128]	8
Number of steps	N_steps_	[3, 10]	7
Relaxation parameter	Γ	[1.0, 2.0]	1.43
Sparsity regularization	Λ	[1 × 10^−6^, 0.01]	1.0 × 10^−6^
Learning rate	H	[1 × 10^−4^, 0.01]	0.0031
Batch size	B	-	32
Virtual batch size	B_v_	-	32

**Table 4:** Evaluation of the different models.

Models	MAE	RMSE	R^2^
RF	0.0421	0.0535	0.7583
RBF	0.0443	0.0490	0.8020
BP	0.0329	0.0440	0.8410
GBDT	0.0336	0.0432	0.8460
XGBoost	0.0338	0.0427	0.8535
TabNet	0.0216	0.0316	0.9246
**TabNet-BO**	**0.0146**	**0.0168**	**0.9681**

**Table 5:** Statistical significance test results between TabNet-BO and baseline models.

Comparison Pair	T-Statistic	*p*-Value
TabNet-BO vs. TabNet	6.027	2.88 × 10^−8^
TabNet-BO vs. BP	7.703	1.04 × 10^−11^
TabNet-BO vs. XGBoost	8.438	2.75 × 10^−13^
TabNet-BO vs. RBF	8.926	2.40 × 10^−14^
TabNet-BO vs. RF	9.602	8.07 × 10^−16^
TabNet-BO vs. GBDT	9.641	6.65 × 10^−16^

## Data Availability

The original contributions presented in the study are included in the article; further inquiries can be directed to the corresponding author.
